# Impact of HPV Types and Dendritic Cells on Recurrent Respiratory Papillomatosis’ Aggressiveness

**DOI:** 10.3390/diseases13020043

**Published:** 2025-02-03

**Authors:** Ellen Eduarda Fernandes, Maria Leticia de Almeida Lança, Yan Aparecido de Souza, Vivian Narana El-Achkar, Victor Costa, Román Carlos, Alfredo Ribeiro-Silva, Laura Sichero, Luisa Lina Villa, Jorge Esquiche León, Estela Kaminagakura

**Affiliations:** 1 Department of Bioscience and Oral Diagnosis, Institute of Science and Technology, São Paulo State University—UNESP, São José dos Campos 12245-000, Brazil; ellen.fernandes@unesp.br (E.E.F.); leticia.lanaa@unesp.br (M.L.d.A.L.); aparecido.souza@unesp.br (Y.A.d.S.); vivian.narana@unesp.br (V.N.E.-A.); victor.costa@unesp.br (V.C.); 2 Centro Clínico de Cabeza y Cuello, Guatemala City 01010, Guatemala; monchorcb@yahoo.com; 3 Department of Pathology and Forensic Medicine, Ribeirão Preto Medical School, Universidade de São Paulo, Ribeirão Preto 14049-900, Brazil; arsilva@fmrp.usp.br; 4 Center for Translational Research in Oncology, Instituto do Cancer do Estado de São Paulo, Hospital das Clinicas da Faculdade de Medicina da Universidade de São Paulo, São Paulo 01246-903, Brazil; laura.sichero@hc.fm.usp.br (L.S.); l.villa@hc.fm.usp.br (L.L.V.); 5 Department of Radiology and Oncology, Faculdade de Medicina, Universidade de São Paulo, São Paulo 01246-903, Brazil; 6 Oral Pathology, Department of Stomatology, Public Oral Health and Forensic Dentistry, School of Dentistry of Ribeirão Preto, University of São Paulo, Ribeirão Preto 14040-904, Brazil; jleon@forp.usp.br

**Keywords:** laryngeal papillomatosis, juvenile laryngeal papillomatosis, human papillomavirus virus, inflammatory cells, dendritic cells

## Abstract

Objective: This study assesses the associations between dendritic cells, HPV 6 and 11, and Recurrent Respiratory Papillomatosis (RRP) aggressiveness. Methods: The Derkay score was calculated using information obtained from the medical records. Biopsies from 36 patients with juvenile RRP (JRRP) and 43 adult RRP (ARRP) patients were analyzed under light microscopy, and their clinical data were collected. Immunohistochemical analysis using antibodies against CD83, CD1a, Factor XIIIa, and S100 was performed, and inflammatory cells were quantified. Data obtained were analyzed using the chi-squared test, in addition to the Mann–Whitney and Z tests for two proportions, considering a confidence interval of 95% and *p* < 0.05 as statistically significant. Results: A higher quantity of S100 was identified in the epithelium (*p* < 0.001) and in the conjunctive tissue (*p* = 0.027) among the ARRP cases, while CD83 (*p* = 0.025) and Factor XIIIa (*p* = 0.018), both in the epithelium, were identified among the JRRP cases. We observed significant association between a higher quantity of CD83 in the epithelium in the juvenile group with a low Derkay index (*p* = 0.034) and with HPV 6 (*p* = 0.039). Conclusions: An increased quantity of dendritic cells is present in individuals diagnosed with RRP, regardless of age, and this may be related to the lower Derkay index, regardless of the HPV type detected.

## 1. Introduction

Recurrent respiratory papillomatosis (RRP) is a benign neoplasm originating from epithelial cells, characterized by outward growth, and linked to low-risk HPV 6 or HPV 11 infection [1]. Despite variations in the prevalence of HPV 6 (B1 and B3) and 11 (A2) genetic variants, these were not significantly associated with atypia [2].

RRP can be categorized based on the onset of disease development, whether before or after 16 years of age, into juvenile (JRRP) or adult (ARRP), respectively [3]. The course of RRP is unpredictable, ranging from spontaneous remission to aggressive growth, leading to airway compromise and often associated with multiple surgical interventions [4], recurrence [5], and risk of malignant transformation [6]. However, the reasons behind the more aggressive course of RRP in some cases remain unclear [7].

Tissue microenvironment alterations have been reported which consequently impact immune response [8]. The infiltration of neutrophilic cells may contribute to local immunosuppression in the papilloma microenvironment [9]. A high frequency of neutrophils can inhibit the activity of T cells, which are the main mediators of cell-mediated immune response. There is a correlation between a higher quantity of CD15 cells and its aggressiveness, as indicated by the Derkay score [10].

Dendritic cells (DCs) are specialized antigen-presenting cells that play a crucial role in innate immunity, acting as a bridge to the adaptive immune system [11,12]. DCs are initiators and modulators of the immune response [13] and regulate immune homeostasis [11]. Depending on their function, maturation level, and location, DCs can exhibit distinct phenotypes [14].

Langerhans cells (LCs) are a subtype of DCs specialized in capturing, transporting, processing, and presenting antigens to T cells. In RRP, immature LCs are characterized by CD1a and langerin (CD207) expression and are furthermore negative for CD14 and CD83 [15]. A higher quantity of mature DCs (CD83) is correlated with more severe RRP cases [16].

The S100 protein has various extra- and intracellular functions [17], playing a key role in several cellular processes such as cell growth and differentiation, in addition to the regulation of innate immune responses [18]. Similar to observations in some malignant pathologies, low-risk HPVs can alter cellular processes [19], such as increasing the presence of growth and angiogenic factors [18], which may modify the expression of S100 protein [19]. Interstitial dendritic cells, represented by Factor XIIIa, are important for immunoglobulin secretion and B cell growth, stimulating antibody production [20].

Due to the critical role of the immune system, especially the dendritic cells (DCs), in managing pathological conditions, and given the unpredictable nature of both juvenile and adult RRP progression, this study explored the potential associations among dendritic cells (DCs), characterized by CD83, CD1a, Factor XIIIa, and S100 expression, HPV type, and disease aggressiveness. Aggressiveness was measured using the Derkay Index, a standardized tool for assessing the severity and clinical impact of RRP. The goal is to improve the early identification of aggressive cases to minimize adverse outcomes.

## 2. Materials and Methods

The study protocol was approved by the Research Ethics Committee of São Paulo State University, approval number: 1.419.232.

### 2.1. Samples

Samples were obtained from three different centers—Pediatric Otorhinolaryngology Outpatient Clinic of the Escola Paulista de Medicina/UNIFESP, Faculty of Medicine of Ribeirão Preto/USP, and Centro Clinico de Cabeza y Cuello in Guatemala—from 1997 to 2016. In total, this study included 56 ARRP samples (46 from Brazil and 10 from Guatemala) and 36 JRRP samples (27 from Brazil and 9 from Guatemala).

Paraffin-embedded blocks of biopsies or surgical specimens and patient data from medical records and reports were available for all samples included in the study. The following data were collected from these medical records: sex, age at diagnosis, lesion location, recurrences, signs and symptoms, and adjuvant treatments.

Patients aged ≤16 years at diagnosis were included in the JRRP group and those >16 years in the ARRP group [3,21]. Derkay laryngoscopic scores [22] were calculated, and samples were grouped according to the level of severity, with scores ≥20 indicating higher severity and scores <20 indicating lower severity [23].

### 2.2. Construction of the Tissue Microarray (TMA)

After a morphological review, regions with representative areas for inflammation in the epithelial (E) and underlying connective (C) tissues were demarcated. Biopsies were then performed from these areas using a manual device or arrayer (Beecher Instrumedics, Microarray Technology, Sun Prairie, WI, United States). Sections of the recipient block were transferred using commercial adhesive film (Instrumedics, Hackensack, NJ, USA) to commercial slides with adhesive (Starfrost@, Instrumedics) and/or positively charged slides (Superfrost Plus@, Erviegas, Alameda Plutão, Brazil) for subsequent fixation by exposing the slides to ultraviolet light for 15 min. The plastic adhesive was removed using a non-chlorinated solvent (TPC@, Instrumedics). The slides were then identified and stored at −20 °C [10].

### 2.3. Immunohistochemistry

Slides were deparaffinized, rehydrated in alcohol, and washed twice in 6% hydrogen peroxide, 20 volumes, for 20 min each at room temperature, followed by washing in 10 mM phosphate-buffered saline (pH 7.4) for 5 min to inhibit endogenous peroxidase. Antigen retrieval was performed in a Pascal pressure cooker (DakoCytomation Denmark A/S. Glostrup, Denmark) in 10 mM citric acid solution (pH 6.0) at 125 °C for 4 min.

Primary antibodies against CD83, CD1a, Factor XIIIa, and S100 were incubated at the dilutions and times indicated in Supplementary Table S1. Reactions were conducted using the EnVision system (Dako Cytomation, Denmark) and visualized using the DAB chromogen (diaminobenzidine, Dako Cytomation, Denmark). Positive and negative controls were included in all reactions, and the sections were counterstained with Mayer’s hematoxylin.

### 2.4. Cells Quantification

TMA slides were digitized using the Pannoramic DESK device (3DHistech^®^, Budapest, Hungary). Without knowledge of the group and sample distinction, blind counting of membranous and/or cytoplasmic and/or nuclear staining was quantified by the researchers (VNRE and MLAL) using the Pannoramic Viewer software 1.15.4 (3DHistech^®^, Budapest, Hungary), considering five representative areas of the lesion, magnified 40×. For the results, the numbers obtained from the average of this counting were used [24]. DCs were considered to be immunopositive cells located above the basal layer of the epithelial tissue.

### 2.5. Statistical Analysis

For the statistical analysis, the SPSS V26 (Somers, NY, USA), Minitab 21.2 (State College, PA, USA), and Excel Office 2010 software were used. Differences were evaluated using the chi-squared test and correlated using the Mann–Whitney and Z tests for Two Proportions. A confidence interval of 95% and results with *p* < 0.05 were considered statistically significant.

## 3. Results

### 3.1. Comprehensive Analysis of Quantitative Factors

The quantitative analysis of all the cases combined, without group distinction, showed a mean age of 23.7 ± 4.4 years for the study participants. Upon analyzing the distribution of qualitative factors such as Derkay (*p* < 0.001), sex (*p* < 0.001), recurrence (*p* = 0.516), tracheostomy (*p* < 0.001), death (*p* < 0.001), location (*p* < 0.001), atypia (*p* < 0.001), HPV 6 (*p* < 0.001), and HPV 11 (*p* = 0.003), the results indicate that there are statistically significant differences in all the factors analyzed, except for recurrence (Table 1).

**Figure 1:**
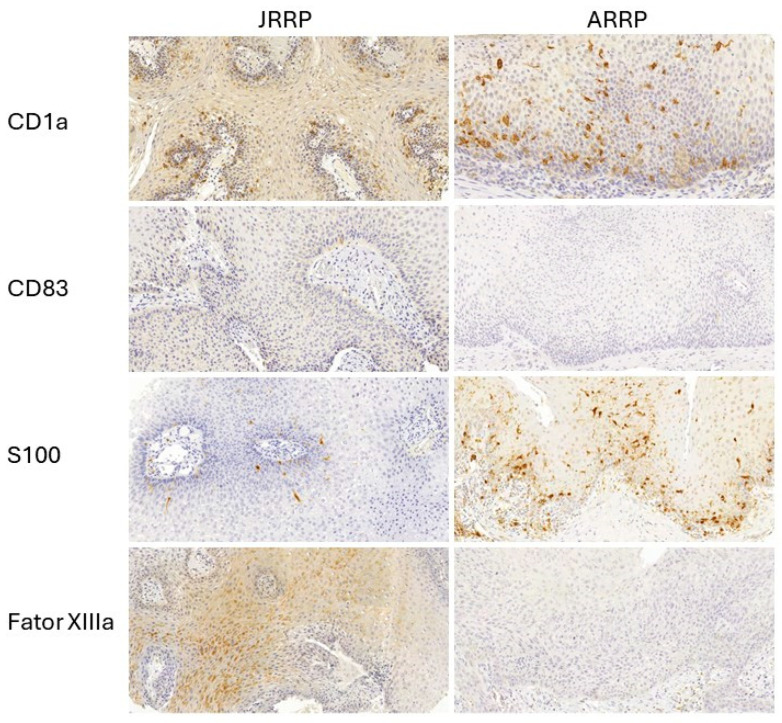
CD1a. Microphotograph of JRRP showing few CD1a cells in the epithelium. CD1a cells dispersed in the epithelium of ARRP. CD83. Negative expression of CD83 in a juvenile sample. Negativity of CD83 in the epithelium of the ARRP group. S100. Few S100+ cells in JRRP, while these cells were abundant in ARRP. Factor XIIIa. Factor XIIIa was observed in epithelial cells of JRRP. Factor XIIIa was negative in ARRP. (DAB stain, Mayer’s counterstain. Microphotograph by Software Pannoramic Viewer 1.15.4, 20× magnification).

Regarding the mean number of inflammatory cells identified within the connective and epithelial tissues, there was a higher prevalence of CD1a (1.33 ± 0.17) and S100 (2.04 ± 0.46) in the epithelium, as well as of Factor XIIIa (6.63 ± 1.49) and CD83 (2.48 ± 0.61) in the connective tissue, regardless of age (Figure 1).

### 3.2. Analysis of Qualitative Factors Comparing JRRP and ARRP Groups

Clinical and demographic data collected from JRRP and ARRP cases are presented in Table 2. There was a higher prevalence of males in the ARRP group at 79.1% (*n* = 34). The assessment of qualitative factors indicated a high incidence of atypia in both groups, particularly in JRRP, where it was present in 100% of the 36 cases. The Derkay index was low for most individuals in the study, including 86.1% (*n* = 31) of JRRP patients and 97.7% (*n* = 42) of ARRP patients.

Concerning the presence of HPV, similar prevalences of HPVs 6 and 11 were observed among the JRRP cases, whereas among the ARRP cases a predominance of HPV 6 (79.1%) was observed. Recurrence of disease was reported for 57.6% (*n* = 19) of individuals in the JRRP group and 48.8% (*n* = 21) in the ARRP group. The tracheostomy rate differed between the groups, with 18.2% (*n* = 6) of JRRP patients and only 2.4% (*n* = 1) of ARRP patients undergoing this procedure. Solitary lesions were present in 63.9% (*n* = 23) and 65.1% (*n* = 28) of JRRP and ARRP patients, respectively.

### 3.3. Analysis of Quantitative Factors

In the epithelial tissue of the JRRP biopsies, a greater quantity of CD83 cells was found when HPV6 (*p* = 0.039) was detected and the Derkay index (*p* = 0.034) was low. Additionally, when Factor XIIIa was more prevalent in connective tissue, there were fewer cases for which disease recurrence was reported (*p* = 0.045).

**Figure 2:**
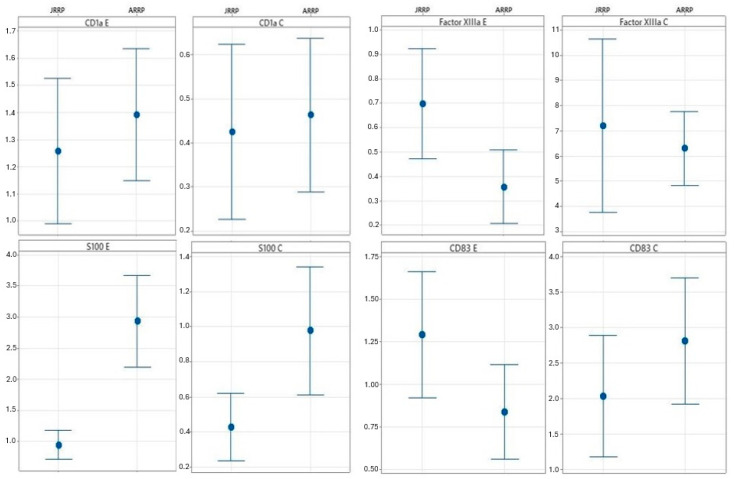
Comparison of the average number of inflammatory cells between groups. (CI) Confidence Interval.

When comparing JRRP and ARRP cases, we observed a higher expression of Factor XIIIa (*p* = 0.018), S100 (*p* < 0.001) and CD83 (*p* = 0.025) in the epithelium of ARRP cases, as well as higher levels of S100 within the connective tissue (*p* = 0.027) (Table 3). The mean of the inflammatory cells found in each group, with a 95% confidence interval, is depicted in Figure 2.

## 4. Discussion

Laryngeal papillomatosis is an HPV manifestation and is associated with types 6 and 11 [25,26,27]. In the present study, we observed that all individuals included presented were detected with at least one of the HPV types, 6 or 11, similar to Makiyama’s et al. [28] results. HPV type 6 was the most frequent [29,30], but HPV6 and HPV11 were found in co-infection in four patients. It has been described that HPV 11-infected RRP cases tend towards greater aggressiveness [31,32].

In this study, the JRRP group presented a higher percentage of tracheostomy (18.2%) and recurrence (57.6%), similar to the findings of Fortes et al. [33] and Wang et al. [34]. HPV infection since childhood can lead to inadequate cellular functioning and compromise the immune system, potentiating the severity of RRP [35], especially among children and adolescents. The immature immune system may reduce antigen processing and/or presentation, as well as attenuate the secretion of pro-inflammatory cytokines, contributing to the severity of the disease [10].

As immature DCs present in the epithelium, when these interact with antigens that bind to cell surface receptors on the membrane, they internalize them into the cellular cytoplasm for processing. As a result of this interaction, DCs undergo maturation and migrate from the skin through the lymphatic vessels to the peripheral lymph nodes, where antigens are presented to T cells, inducing both immune responses [36]. In inflammation, DC precursors migrate from the blood to the tissue, increasing the local cellular quantity [37].

The immune system plays a crucial role in the outcome of HPV infection [16]. DCs initiate a cellular immune response responsible for eliminating virus-infected keratinocytes [38]. Mature DCs (CD83) play an essential role in establishing immunological crosstalk with T cells [39]. In our study, we observed a higher quantity of CD83 in the epithelial tissue in the JRRP group associated with HPV 6 but not with aggressiveness. However, Kovalenko et al. [16] identified a higher quantity of CD83 cells in JRRP and correlated this with the severity of the disease.

In this study, CD1a expression did not differ significantly between the evaluated groups, consistent with the findings of Kovalenko et al. [16]. Similarly, in human immunodeficiency virus (HIV) and herpes simplex virus type 2 infections, CD1a cell numbers remained stable, reflecting the persistence of Langerhans cell populations during viral infections [40].

The ARRP group showed higher S100 expression in both the epithelial and conjunctive tissues, similar to the findings of DeVoti et al. [18]. S100 cells play a role in regulating the innate immune response to pathogens [18], being important pro-inflammatory factors of innate immunity [41]. The members of the S100 family act in the immune system as alarm signals (damage-associated molecular patterns—DAMPs), chemoattractants, pro-inflammatory stimulators, regulators of immune cells, as well as antimicrobial peptides and metal eliminators during an innate immune response [42]. The increased density of Langerhans cells (LCs) in RRP is often associated with increased lymphoid infiltrate in the epithelial tissue and stroma, indicating that local immunity is not only preserved but intensified [43]. On the other hand, the overall reduction in CD1a, CD83, and Factor XIIIa cells in the lymphoid tissues of individuals with HIV may indicate a loss of disease control by the immune system [14].

The presence of mature DCs has been correlated with a positive prognosis in ovarian carcinoma [44], and there is a positive association between DC presence and patient survival in different types of cancer, such as metastatic melanoma, breast cancer, head and neck squamous cell carcinoma, and lung adenocarcinoma [45]. Moreover, an increase in the number of dendritic cells has been associated with a better disease-free survival rate for patients with melanoma [46]. Similarly, a higher number of CD83 and Factor XIIIa cells was observed in the JRRP group, as well as S100 cells in the epithelial and conjunctival tissues of the ARRP group, along with a higher value of Factor XIIIa, regardless of age and associated with a low Derkay index.

## 5. Conclusions

In conclusion, the increased values of CD83, S100, and Factor XIIIa in patients with RRP suggest a more efficient local immunogenicity, which appears to be associated with lower aggressiveness.

## Figures and Tables

**Table 1:** Qualitative results from the samples analyzed collectively, without group distinction.

		*n*	%	*p*
Group	ARRP	43	54.4	0.265
	JRRP	36	45.6	
Derkay	High	6	7.6	<0.001
	Low	73	92.4	
Sex	Female	27	34.2	<0.001
	Male	52	65.8	
Recurrence	No	36	47.4	0.516
	Yes	40	52.6	
Tracheostomy	No	67	90.5	<0.001
	Yes	7	9.5	
Death	No	76	96.2	<0.001
	Yes	3	3.8	
Location	1	51	64.4	<0.001
	≥2	28	35.4	
Atypia	Absent	5	6.3	<0.001
	Present	74	93.7	
HPV 6	Absent	26	32.9	<0.001
	Present	53	67.1	
HPV 11	Absent	49	62.0	**0.003**
	Present	30	38.0	

*p* values in bold are statistically significant.

**Table 2:** Distribution of qualitative factors in JRRP and ARRP cases.

		JRRP		ARRP		*p*
		*n*	%	*n*	%	
Sex	Female	18	50.0	9	20.9	**0.007**
	Male	18	50.0	34	79.1	
Atypia	Absent	0	0.0	5	11.6	**0.035**
	Present	36	100	38	88.4	
Derkay	High	5	13.9	1	2.3	0.053
	Low	31	86.1	42	97.7	
HPV 11	Absent	17	47.2	32	74.4	**0.013**
	Present	19	52.8	11	25.6	
HPV 6	Absent	17	47.2	9	20.9	**0.013**
	Present	19	52.8	34	79.1	
Death	No	35	97.2	41	95.3	0.664
	Yes	1	2.8	2	4.7	
Recurrence	No	14	42.4	22	51.2	0.450
	Yes	19	57.6	21	48.8	
Tracheostomy	No	27	81.8	40	97.6	**0.021**
	Yes	6	18.2	1	2.4	
Number of RRP	1	23	63.9	28	65.1	0.535
	≥2	13	36.1	15	34.9	

*p* values in bold are statistically significant.

**Table 3:** Comparison of age and inflammatory cells in epithelial and conjunctive tissues between groups.

	Group	Mean	Median	SD *	*p* ^#^
CD1a E	JRRP	1.26	1	0.78	0.367
	ARRP	1.39	1	0.77	
CD1a C	JRRP	0.42	0	0.56	0.724
	ARRP	0.46	0	0.55	
Factor XIIIa E	JRRP	0.70	1	0.64	**0.018**
	ARRP	0.36	0	0.48	
Factor XIIIa C	JRRP	7.20	4	8.31	0.823
	ARRP	6.30	5	4.75	
S100 E	JRRP	0.94	1	0.68	**<0.001**
	ARRP	2.93	2	2.39	
S100 C	JRRP	0.43	0	0.56	**0.027**
	ARRP	0.98	1	1.18	
CD83 E	JRRP	1.29	1	1.01	**0.025**
	ARRP	0.84	1	0.90	
CD83 C	JRRP	2.03	2	2.28	0.092
	ARRP	2.80	2	2.80	

*p* values in bold are statistically significant; * Standard Deviation; ^#^ *p*-valor ≤ 0.05.

## Data Availability

The original contributions presented in this study are included in the article/supplementary material. Further inquiries can be directed to the corresponding author.

## Supplementary Materials

The following supporting information can be downloaded at: https://www.mdpi.com/article/10.3390/diseases13020043/s1, Table S1: List of primary antibodies.
