# Supplementary material for: Impact of HPV Types and Dendritic Cells on Recurrent Respiratory Papillomatosis’ Aggressiveness
Source: Diseases. 2025 Feb 3;13(2):43. doi: 10.3390/diseases13020043 (PMC11854725; doi:10.3390/diseases13020043)
Supplement: Supplementary file 1 [file diseases-13-00043-s001.zip › diseases-3367661-supplementary.pdf]

**Antibody**	**Dilution**	**Clone**	**Manufacturer**
CD1a	1:400	010	DakoCytomation, Glostrup, Denmark
Factor XIIIa	1:100	E980.1	Leica Biosystems Newcastle, Ltd
S100	1:1000	Polyclonal	Leica Biosystems Newcastle, Ltd
CD83	1:100	1H4b	Leica Biosystems Newcastle, Ltd
